# The Contemporary Role of Photodynamic Therapy in the Treatment of Pachychoroid Diseases

**DOI:** 10.1155/2021/6590230

**Published:** 2021-10-23

**Authors:** Lawrence P. L. Iu, Ho Yan Chan, Mary Ho, Frank H. P. Lai, Andrew C. Y. Mak, Raymond L. M. Wong, Alvin L. Young

**Affiliations:** ^1^Department of Ophthalmology and Visual Sciences, Prince of Wales Hospital, Shatin, Hong Kong; ^2^Department of Ophthalmology and Visual Sciences, The Chinese University of Hong Kong, Shatin, Hong Kong; ^3^Department of Ophthalmology, Caritas Medical Centre, Sham Shui Po, Hong Kong

## Abstract

Recent advances in retinal imaging technology have improved our understanding in the pathogenesis and evolvement of various chorioretinal diseases. Central serous chorioretinopathy and polypoidal choroidal vasculopathy are now recognized to belong to the same spectrum of disorders known as pachychoroid diseases. Pachychoroid diseases have similar pathogenesis pathway and common characteristics of thickened choroid, dilated outer choroidal vessels, and thinning of choriocapillaris. More disease entities have been identified to belong to this disease spectrum. Photodynamic therapy can induce choroidal hypoperfusion, remodeling of abnormal choroidal vessels, and reduction of choroidal congestion. It is known to be an effective treatment for chronic central serous chorioretinopathy and polypoidal choroidal vasculopathy. Many new studies are being performed to investigate its efficacy in other pachychoroid diseases. In this review, we provided an overview of the rationale, efficacy, and treatment strategies of photodynamic therapy in different pachychoroid diseases and discussed its role in the management along with other treatment modalities with most updated clinical evidence.

## 1. Introduction 

Recent advances in retinal imaging technology over the past decade have allowed detection of fine abnormalities in different chorioretinal conditions and facilitated evaluation of the anatomical location of diseases in various choroidal and retinal layers. This has improved our understanding in the pathogenesis and evolvement of various chorioretinal diseases. In particular, advents of enhanced-depth imaging of optical coherence tomography (OCT) and swept-source OCT, which allow deeper penetration of signals beyond the retinal layers, helped in the study of choroidal layers in better detail. [1, 2] With the introduction of OCT angiography, investigators can now easily evaluate and monitor the characteristics of abnormal vessels and neovascularization in various choroidal vascular diseases [2, 3].

Central serous chorioretinopathy (CSC) and polypoidal choroidal vasculopathy (PCV) have long been known to have abnormal choroidal vascular hyperpermeability in indocyanine green angiography (ICGA) [4–6]. Only recently did we recognize that both CSC and PCV belong to a common spectrum of disorders known as pachychoroid diseases, with more entities now being identified to belong to this spectrum [7, 8]. Photodynamic therapy (PDT) has been shown in many studies to be an effective treatment for chronic CSC through the mechanism of inducing choroidal hypoperfusion, choroidal vessel remodeling, and reduction of choroidal congestion [9]. It would be intriguing to know if PDT would also be effective for other pachychoroid diseases with similar pathogenesis pathway. This does not only contain important therapeutic values, but it also provides insights into the etiologies and pathogenesis of various pachychoroid diseases.

A literature search was conducted using PubMed database for articles related to the efficacy of PDT in treating different pachychoroid diseases. The search was limited to English articles, with full text only and published up to July 2021. The aim of this review is to provide an overview of the rationale, efficacy, and strategies of PDT in different pachychoroid diseases and to discuss its role in the management along with other treatment modalities with most updated clinical evidence.

## 2. Pachychoroid Disease

Pachychoroid disease (or pachychoroid spectrum disorders) refers to a spectrum of diseases which have the common characteristics of diffuse or focal choroidal thickening, dilated outer choroidal vessels in Haller's layer (pachyvessels) with compression and attenuation of inner choroidal vessels at Sattler's layer and choriocapillaris [7, 8]. Six disease entities have been identified so far to belong to this spectrum, namely, CSC, PCV, pachychoroid pigment epitheliopathy (PPE), pachychoroid neovasculopathy (PNV), focal choroidal excavation (FCE), and peripapillary pachychoroid syndrome (PPS) [7, 8]. These entities are believed to share a common pathogenesis pathway, which results in outer choroidal vascular dilatation, choroidal vascular hyperpermeability, and disruption of retinal pigment epithelium (RPE) [2, 7, 8].

The exact etiology of pachychoroid disease is unknown, but recent evidence using wide-field ICGA and OCT suggested that it is related to the formation of intervortex venous anastomosis at the watershed area in the posterior pole [10–12]. Intervortex venous anastomosis is more commonly seen in patients with pachychoroid diseases than in normal individuals [10]. These anastomotic venous channels are believed to be the origin of pachyvessels [10].

## 3. Photodynamic Therapy: Protocol and Mechanism of Action

PDT is a form of phototherapy that requires components of light radiation and photosensitizing agent. [13] The photosensitizing agent is applied and accumulates in the target pathological tissues [13]. Upon irradiation of light with particular wavelengths, the photosensitizing agent will be activated to an excited state and forms free radicals and singlet oxygen, thereby destroying the pathological tissues selectively. PDT has been widely used to treat many oncological and dermatological diseases [13].

In the eye, PDT is mainly used to treat abnormal chorioretinal vascular diseases and tumors [14, 15]. The photosensitizing agent used is verteporfin which has a predilection for the low-density lipoprotein receptors expressed on abnormal vascular endothelial cells [14]. The light irradiation used is a near-infrared light with a typical wavelength of 689 nm [14, 15]. After activation by light, verteporfin produces a photochemical reaction and induces thrombosis and occlusion of the abnormal vessels [14]. The abnormally dilated choroidal vessels in pachychoroid diseases make it a favorable target for PDT to work on.

The standard protocol for ocular PDT involves intravenous infusion of verteporfin at a dose of 6 mg/m^2^ body surface area (BSA) over 10 minutes [14, 16]. At 15 minutes after initiation of infusion, a near-infrared light with wavelength 689 nm is applied to the targeted region for 83 seconds at a fluence rate of 50 J/cm^2^ through a laser contact lens [14, 16]. The light irradiation is applied when verteporfin is most concentrated in the target pathological tissue relative to surrounding normal tissue [14]. To enhance treatment safety, protocols with lower treatment energy or shorter duration have been developed [16]. These include half-fluence PDT, where a lower light energy setting of 25 J/cm^2^ is used instead of 50 J/cm^2^, half-dose PDT, where a lower dosage of verteporfin at 3 mg/m^2^ BSA is used instead of 6 mg/m^2^ BSA, and half-time PDT, where the duration of light irradiation is halved compared to standard protocol [16].

The mechanism of PDT in pachychoroid diseases involves promotion of choriocapillaris hypoperfusion, which in turn reduces choroidal congestion, improves choroidal hyperpermeability, and decreases extravascular leakage [16].

## 4. Efficacy of Photodynamic Therapy in Different Pachychoroid Diseases

### 4.1. Central Serous Chorioretinopathy

CSC is a chorioretinal disease characterized by focal or multifocal serous retinal detachment at the macula due to choroidal vascular hyperpermeability and RPE dysfunction [9]. Many studies reported an association between CSC and certain personality traits and mental disorders [9, 17, 18]. While many CSC resolve spontaneously within 3-4 months, a subgroup of patients have persistent and recurrent subretinal fluid (SRF) leading to permanent RPE and photoreceptors damage and retinal atrophy [9]. Chronic CSC is generally defined as persistence of SRF for more than 4–6 months and presence of atrophic changes in the retina and RPE [9].

Among the available treatment options, PDT is considered to be most efficacious in treating chronic CSC [19]. PDT was first demonstrated as an effective treatment for chronic CSC in 2003 [20]. Choroidal vascular remodeling, normalization, and reduction of extravascular leakage were observed as early as 1 month after treatment. [20] Potential complications, however, may occur after PDT, and they include choroidal ischemia, RPE atrophy, and development of choroidal neovascularization (CNV) [21, 22]. Safety-enhanced protocols with half-dose (3 mg/m^2^ BSA) or half-fluence (25 J/cm^2^) PDT were, therefore, developed to reduce adverse effects [21, 23]. These safety-enhanced PDT protocols were shown to be safe and effective in improving visual acuity (VA) and central retinal thickness (CRT) in chronic CSC in many studies [24–27].

Currently, either half-dose or half-fluence PDT is the standard of treatment for chronic CSC. Further reduction in treatment energy with half-dose-half-fluence PDT has not been shown to be effective in improving VA or CRT when compared to half-dose or full-dose PDT [28]. When comparing half-dose versus half-fluence PDT, Nicolo et al. [29] showed that the treatment effect of half-dose PDT was more rapid and more long-lasting than half-fluence PDT in a retrospective study of 56 patients. The proportion of patients with complete SRF resolution was significantly higher in the half-dose PDT group than the half-fluence PDT group at 1 month (86% versus 61%) and 12 months (100% versus 83%) after treatment [29]. However, other studies did not show significant differences in the efficacy between half-dose and half-fluence PDT in treating chronic CSC. In a retrospective study of 60 patients, Alkin et al. [27] showed no significant differences in the proportion of complete SRF resolution (92% versus 91%) and VA improvement (+4.8 letter versus +7.4 letters) between half-dose and half-fluence PDT at 1 year. Kim et al. [30] also showed no significant difference in the proportion of complete photoreceptor recovery with continuous ellipsoid zone in OCT (54% versus 73%) between half-dose and half-fluence PDT at 1 year.

Half-time PDT has been shown to be safe and effective for chronic CSC in several retrospective studies [31, 32] Liu et al. [33] compared the result of half-time versus half-dose PDT in chronic CSC and showed that there were no significant differences in terms of VA improvement (−0.15 logMAR units versus −0.25 logMAR units) and proportion of complete SRF resolution (100% versus 91%) at 1 year. Similarly, Peng et al. [34] showed no significant differences between half-time and half-dose PDT in terms of VA gain (−0.17 logMAR units versus −0.11 logMAR units) and proportion of complete SRF resolution (94% versus 94%) at 1 year.

Half-dose PDT was shown to be more effective than subthreshold micropulse laser (MPL) in treating chronic CSC in a large randomized controlled trial. The PLACE trial [35] was a multicenter, randomized controlled trial, in which 179 patients with chronic CSC were randomized in a 1 : 1 ratio to receive half-dose PDT versus high-density subthreshold MPL. The study showed that, at 7-8 months, half-dose PDT was superior to MPL in achieving complete SRF resolution (67.2% versus 28.8%) and improvement in retinal sensitivity on microperimetry (+3.24 dB versus +1.38 dB). The VA improvement was significantly better after half-dose PDT than after MPL at 6–8 weeks (+4.60 letters versus +1.39 letters), but the difference was not statistically significant at 7-8 months (+6.78 letters versus +4.48 letters). Similarly, in our previous study, [36] we showed that patients receiving half-dose PDT had faster recovery of choriocapillaris flow deficit on OCT angiography (as early as 3 months versus 6 months), better chance of complete SRF resolution (87% versus 50% at 3 months), and greater reduction in foveal choroidal volume (−47 mm^3^ versus +25 mm^3^) compared to MLT, suggesting that half-dose PDT was superior to MLT in promoting choriocapillaris recovery.

PDT was shown to be superior to intravitreal anti-VEGF in chronic CSC. Bae et al. [37] showed in a randomized controlled trial of 34 eyes that half-fluence PDT was significantly better than 3 monthly injections of ranibizumab in achieving complete SRF resolution (89% versus 12%) and reduction in ICGA choroidal vascular hyperpermeability (89% versus 0%) at 1 year. Half-fluence PDT also resulted in better VA compared to ranibizumab at 3 months (0.13 logMAR units versus 0.24 logMAR units), but the difference was not statistically significant at 1 year (0.12 logMAR units versus 0.17 logMAR units).

### 4.2. Polypoidal Choroidal Vasculopathy

PCV was first recognized and reported in 1990s [38]. It is characterized by the presence of abnormal branching vascular network (BVN) with aneurysmal dilatation of vessels in a polypoidal configuration [39]. PCV was initially considered to arise from abnormal choroidal vessels and was diagnosed mainly by ICGA [38]. Advances in imaging technologies including OCT angiography showed that PCV was in fact an aneurysmal dilatation of type 1 (sub-RPE) neovascularization in the context of pachychoroid [7, 40]. The neovascular network, aneurysmal lesions, and feeding vessels were all shown to be located anterior to Bruch's membrane [7, 40].

Before the emergence of intravitreal anti-VEGF agents, PDT was the mainstay of treatment for macular PCV. The efficacy of PDT was shown in many early studies [5]. However, the improvement and stabilization of VA were short-term only. Recurrence was common due to persistent BVN and development of new active lesions after PDT [5]. A retrospective study with long-term follow-up of 68 eyes [41] showed that VA remained stable for 2 years after PDT, but it became significantly worse at 3 years. The cumulative recurrence rate of PCV was 16%, 34%, and 52% at 1, 2, and 3 years, respectively, after treatment, and recurrence was significantly associated with VA loss of 15 letters or more [41]. Severe complications may also occur: subretinal hemorrhage, RPE tear, and massive suprachoroidal hemorrhage have been reported after PDT for PCV [5, 42, 43].

When intravitreal anti-VEGF agents became available, interests were switched from PDT to intravitreal anti-VEGF injections in the treatment for PCV and neovascular AMD [44]. The safety and efficacy of PDT monotherapy or combined with anti-VEGF in comparison to anti-VEGF alone were evaluated in several large, multicentered, pivotal randomized controlled trials: EVEREST I, EVEREST II, and PLANET studies.

In the EVEREST I study [45], 61 patients were randomized in a 1 : 1:1 ratio to receive PDT monotherapy, PDT combined with monthly ranibizumab, or monthly ranibizumab monotherapy. It showed that PDT monotherapy or in combination with ranibizumab had significantly higher rate of complete regression of polypoidal lesion at 6 months compared to ranibizumab alone (71.4% and 77.8% versus 28.6%). However, VA gain occurred in all 3 groups (+7.5 letters in PDT monotherapy, +10.9 letters in combined PDT plus ranibizumab, and +9.2 letters in ranibizumab monotherapy), and there were no significant differences among the three groups. The proportion of patients gaining 15 letters or more in VA was 19% (PDT monotherapy), 21% (PDT plus ranibizumab), and 33% (ranibizumab monotherapy), respectively. In summary, EVEREST I study suggested that PDT was superior to anti-VEGF in achieving complete polypoidal regression, but the visual improvement was similar among PDT, ranibizumab, and combination therapy. The limitations of EVEREST I study were that it did not have long-term results beyond 6 months, and it failed to demonstrate which treatment strategies had better VA gain. EVEREST II study [46] was, therefore, performed to address these questions.

The aim of EVEREST II study was to compare the efficacy of combined PDT plus ranibizumab versus ranibizumab monotherapy in PCV for 24 months. The EVEREST II study included 322 patients and showed that combination therapy resulted in significantly better VA gain (+9.6 letters versus +5.5 letters), higher chance of complete polypoidal lesion regression (56% versus 26%), and fewer number of ranibizumab injections (6.0 versus 12.0) than ranibizumab monotherapy at 24 months after treatment. This study suggested that combination therapy was superior to ranibizumab alone in the treatment for PCV.

PLANET study [47, 48] was a randomized controlled trial similar to EVEREST II study except with the use of aflibercept instead of ranibizumab and the use of PDT as rescue therapy instead of initial treatment. In the PLANET study, 318 patients received 3 monthly injections of aflibercept, and if the treatment response was suboptimal, they were then randomized to receive either aflibercept monotherapy or combined aflibercept plus rescue PDT. This study showed that rescue PDT had no additional benefit, as both aflibercept plus rescue PDT and aflibercept monotherapy groups achieved similar VA gain (+10.7 letters versus +9.1 letters), complete polyp regression rate (33% versus 29%), and polyp inactivation rate (82% versus 85%) at 24 months. The authors concluded that rescue PDT had no additional benefit if aflibercept was used.

The Fujisan study [49] was a randomized controlled trial evaluating whether PDT would be more useful as initial treatment (given within 1 week of first ranibizumab) or deferred treatment (given after 3 monthly ranibizumab injections if necessary) for PCV. This study included 72 patients and showed that both initial and deferred PDT resulted in similar VA gain (+8.1 letters versus +8.8 letters) and polypoidal lesion regression rate (62% versus 54%) at 1 year. However, fewer additional retreatments were required when PDT was given initially (1.5 ranibizumab injections and 0.14 PDT) compared to deferred PDT group (3.8 ranibizumab injections and 0.45 PDT).

Half-dose PDT has been evaluated as treatment for PCV in two prospective studies [50, 51]. One study showed that half-dose PDT combined with ranibizumab could successfully induce polypoidal lesion regression if there was single polyp only without any BVN [50]. The other study showed that half-dose PDT combined with ranibizumab would improve VA significantly at 1 year if the number of polyps was few on ICGA (≤3) and the VA was good (>20/50) at baseline [51].

In summary, clinical studies suggested that intravitreal anti-VEGF was beneficial in improving VA in PCV. Adding PDT seemed to improve the success rate of polypoidal lesion regression and may reduce the required number of intravitreal anti-VEGF injections when ranibizumab was used. The efficacy of half-dose PDT for PCV was limited only, and it should be considered only in selected cases with small number of polypoidal lesions.

### 4.3. Pachychoroid Neovasculopathy

PNV was first described by Pang and Freund in 2015 [52]. PNV is characterized by the presence of type 1 (sub-RPE) neovascularization above regions of choroidal thickening and pachyvessels [52]. PNV can develop from PPE or CSC [53].

The pathogenesis of PNV is not fully understood [53]. It was postulated that the dilated outer choroidal vessels compress on the choriocapillaris and resulted in choriocapillaris ischemia, RPE dysfunction, and hence development of type 1 neovascularization and PNV [53]. The mechanism in which VEGF is involved in causing CNV might be different between PNV and neovascular AMD, as the study showed that the intraocular VEGF level in PNV was significantly lower than that in neovascular AMD [54]. Nonetheless, PNV responded favorably to anti-VEGF treatment [54, 55]. Jung et al. [55] showed that 3 monthly injections of aflibercept or ranibizumab were effective in improving VA, reducing central choroidal thickness (CCT), and reducing CRT in PNV. The reduction of CCT and rate of complete SRF resolution were significantly higher in the aflibercept group than ranibizumab group at 3 months (−35 *µ*m versus −9 *µ*m in CCT reduction and 82% versus 51% in complete SRF resolution) [55]. Recurrence of SRF within 12 months was also less commonly seen in the aflibercept group than ranibizumab group (36% versus 56%), but the difference was not statistically significant [55].

A recent retrospective study compared the result of half-dose PDT versus anti-VEGF treatment with 3 monthly injections of ranibizumab or aflibercept in 82 eyes with PNV [56]. It showed that the percentage of complete SRF resolution was similar between those receiving half-dose PDT and anti-VEGF at 3 months (82% versus 96%) and at 1 year (85% versus 94%). Both groups had significant VA improvement at 3 months, and the VA remained stable for 1 year. On average, patients required 3.7 injections in the anti-VEGF group and 1.1 PDT in the PDT group over 1 year. This study suggested that half-dose PDT was as effective as anti-VEGF treatment for PNV.

Since PNV has components of both pachychoroid and CNV, it is rational to use combination therapy with PDT to treat the choroidal congestion and anti-VEGF to treat the neovascularization in PNV. Combining PDT with anti-VEGF treatment had been evaluated and was shown to be effective for PNV in several retrospective studies. Miki et al. [57] evaluated the result of full-dose PDT combined with one injection of ranibizumab or aflibercept in 42 eyes with PNV and showed that VA, CRT, and CCT all improved significantly and maintained at 12 months. In this study, 76% did not require additional treatments. Karasu and Celebi [58] evaluated the result of full-dose PDT combined with 3 monthly injections of bevacizumab, ranibizumab, or aflibercept in 19 eyes with PNV. Aflibercept appeared to be superior in terms of VA change (−0.18 logMAR units) compared to ranibizumab and bevacizumab (+0.30 logMAR units and +0.43 logMAR units respectively). However, the number of eyes in each group was small, and therefore, whether aflibercept was superior to the other anti-VEGF agents required larger studies to confirm. Matsumoto et al. [59] studied the result of half-fluence PDT combined with one injection of aflibercept in 21 eyes with PNV and showed that VA, CRT, CCT, and CNV thicknesses all improved significantly at 12 months, and 81% of eyes had no recurrence of CNV [59]. Kitajima et al. [60] evaluated the result of full-dose PDT plus 3 monthly injections of ranibizumab in 11 eyes and showed that VA, CRT, and CCT all improved significantly at 1 year, and 54% of eyes did not require retreatment.

### 4.4. Focal Choroidal Excavation

FCE is characterized by a focal area of depression (excavation) in choroid, which can be easily detected by OCT [61, 62]. FCE may appear clinically as hypo- or hyperpigmentary change, yellowish lesion, or even without any obvious changes in the macula [61]. It was first described by Jampol et al. [63] in 2006 and was later classified by Margolis et al. [64] in 2011 into 2 types: conforming and nonconforming. In the conforming type, the outer retinal layer is still attached to RPE and follows the contour of choroidal excavation [64]. In the nonconforming type, the outer tips of photoreceptors are separated from RPE in the excavated region with a potential space in between [61]. The space is considered to contain SRF and appears hyporeflective under OCT. Patients may have symptoms of blurring of vision or metamorphopsia in nonconforming FCE. FCE can be pachychoroid-associated and has been reported in cases with CSC, PCV, and isolated thickened choroid [62]. In addition to pachychoroid, FCE has also been shown to be associated with inflammatory conditions such as multiple evanescent white dot syndrome, multifocal choroiditis and panuveitis, punctate inner choroidopathy and Vogt-Koyanagi-Harada disease; [65–68] and dystrophy conditions such as Stargardt disease, best vitelliform macular dystrophy, and cone dystrophy [69–72].

The exact pathogenesis of FCE is unknown. It is postulated that some cases of FCE are congenital due to developmental defect [61], while others are acquired, which occur as a consequence of contraction of choroidal connective tissue scar leading to outpouching of RPE and choroid towards the sclera after resolution of CNV and other inflammatory processes [61, 73]. Banaee et al. described a case of FCE formation at site of previous PPE, in which the FCE appeared along with the disappearance of the pachyvessel [74]. The authors suggested that vascular thrombosis of pachyvessel could be the mechanism of FCE formation.

Isolated conforming FCE is usually asymptomatic, and treatment is not necessary. However, conversion of conforming to nonconforming FCE with SRF accumulation due to development of CSC could occur [75]. On the other hand, conversion from nonconforming to conforming FCE due to resolution of SRF had also been observed [75]. In a retrospective case series [76] of 116 patients with CSC, 6% were noted to have coexisting FCE (around half were conforming and half were nonconforming) [76]. Half-dose PDT were given to two cases of nonconforming FCE in this series, and the SRF resolved completely with VA improvement. The FCE remained unchanged. Okubo et al. [77] reported a case of PCV with FCE, which was treated with full-dose PDT [77]. The SRF resolved with VA improvement, and the FCE remained unchanged [77].

### 4.5. Pachychoroid Pigment Epitheliopathy

PPE was first described by Warrow et al. in 2013. [78] PPE is characterized by the presence of RPE alternations in the posterior pole above regions of choroidal thickening and pachyvessels [8, 78].

PPE is considered as a precursor of CSC [7]. PPE can occur in isolation or in uninvolved fellow eyes of patients with other pachychoroid diseases [78, 79]. It is common in asymptomatic fellow eyes of patients with unilateral CSC. A retrospective study of 282 patients showed that PPE was present in 61% of fellow eyes with unilateral CSC [80]. A longitudinal study of 46 eyes showed that 17% of eyes with PPE eventually developed CSC after a mean follow-up of 5 years [81]. Since PPE is asymptomatic, and the majority does not develop sight-threatening conditions, observation with serial OCT monitoring is the mainstay of management for isolated PPE, and treatment is not considered necessary unless CSC, PNV, or PCV develops.

### 4.6. Peripapillary Pachychoroid Syndrome

PPS was recognized as a pachychoroid disease entity by Phasukkijwatana et al. in 2018 [82]. PPS has the characteristics of greater choroidal thickening at nasal macula compared with temporal macula, presence of intraretinal fluid (IRF) or SRF in the peripapillary region, and presence of underlying pachyvessels [82]. The features to distinguish PPS from CSC include location at the peripapillary region, choroidal thickening mainly at nasal macula, presence of peripapillary IRF, and clinical appearance of crowded disc [83].

Due to relatively new recognition of this entity, only limited studies had evaluated the result of intervention for PPS. A retrospective study [84] of 25 eyes showed that PDT was effective in reducing SRF and CCT at 3 months, and the effect remained stable at 12 months. VA also improved from 0.59 logMAR units at baseline to 0.51 logMAR units at 3 months; however, the VA gain subsided with time afterwards to 0.60 logMAR units at 6 months and 0.65 at 12 months. Complete SRF and IRF resolution occurred in 40% and 36% of eyes, respectively, at 3 months. The authors concluded that PDT was effective for PPS, but the visual improvement reduced over time. Of note, only 36% of eyes in this study were treatment-naïve, and the rest had failed previous treatments including anti-VEGF injections, oral eplerenone, or MPL. Therefore, these patients might represent a relatively refractory type of PSS. Moreover, only 4% of eyes in this study received full-dose PDT, and the remaining received half-fluence PDT (56%), half-dose PDT (12%), and half-dose-half-fluence PDT (28%). Another retrospective study [83] with long-term follow-up showed that, even without treatment, VA in PPS patients remained stable for 27 months. Therefore, PDT appeared to be a viable treatment option for PPS with anatomical improvement; however, complete SRF and IRF resolution were not achieved in more than half of the patients, and the VA gain was not sustained. In PPS, there is a lack of prospective studies with standardized treatment to confirm the role of PDT.

## 5. Summary

In summary, pachychoroid disease consists of a spectrum of disorders with common choroidal characteristics and structural abnormalities. It appears that PPE resides on one end of this spectrum, and PCV on the other. PPE is asymptomatic, but it may evolve into CSC or PNV in some cases. PNV occurs when type 1 CNV develops, and PCV occurs when type 1 CNV dilates in an aneurysmal configuration. The mechanism and predisposing factors in which one entity evolves into another are yet to be elucidated. The position of PPS in this spectrum is unclear, but it resembles CSC and has a predilection for the peripapillary region. It is uncertain if FCE is the cause or aftermath of pachychoroid diseases [73], as it is also seen in many inflammatory and degenerative eye conditions unrelated to pachychoroid.

In pachychoroid diseases, PDT is beneficial in reducing choroidal vascular hyperpermeability and promoting remodeling of vessels. PDT monotherapy appears to be sufficient for conditions in the more benign end of spectrum like CSC before development of CNV. When type 1 CNV has developed as in PNV and PCV, PDT alone does not appear to be sufficient for visual improvement, and other treatment modalities like intravitreal anti-VEGF become necessary. The potential complications of PDT also limit its repeated use in refractory and recurrent cases. The need of anti-VEGF treatment in combination or adjunction with PDT is expected for diseases towards the neovascular end of spectrum like PNV and PCV. Evidence also suggests that the treatment effect of PDT is dose-dependent on pachychoroid disease. While half-dose PDT is sufficiently effective for CSC, it is not effective for PCV unless the polypoidal lesion is small.

It is useful to consider the different pachychoroid disorders as a continuous process in the pachychoroid disease spectrum [85]. This could help our understanding of disease pathogenesis and in determining the role of PDT and other treatment modalities in pachychoroid disease [86]. Successful management also relies on future researches and investigations about the etiology of pachychoroid, the pathophysiology, how one disease entity evolves into another, optimal effective dose of PDT for each disease entity, and the role of other treatment modalities in the management of pachychoroid disease [7].

## Data Availability

The data supporting this review article are from previously reported studies, which have been cited.
